# PolymiRTS Database 3.0: linking polymorphisms in microRNAs and their target sites with human diseases and biological pathways

**DOI:** 10.1093/nar/gkt1028

**Published:** 2013-10-24

**Authors:** Anindya Bhattacharya, Jesse D. Ziebarth, Yan Cui

**Affiliations:** ^1^Department of Microbiology, Immunology and Biochemistry, University of Tennessee Health Science Center, Memphis, TN 38163, USA and ^2^Center for Integrative and Translational Genomics, University of Tennessee Health Science Center, Memphis, TN 38163, USA

## Abstract

Polymorphisms in microRNAs (miRNAs) and their target sites (PolymiRTS) are known to disrupt miRNA function, leading to the development of disease and variation in physiological and behavioral phenotypes. Here, we describe recent updates to the PolymiRTS database (http://compbio.uthsc.edu/miRSNP), an integrated platform for analyzing the functional impact of genetic polymorphisms in miRNA seed regions and miRNA target sites. Recent advances in genomic technologies have made it possible to identify miRNA–mRNA binding sites from direct mapping experiments such as CLASH (cross linking, ligation and sequencing of hybrids). We have integrated data from CLASH experiments in the PolymiRTS database to provide more complete and accurate miRNA–mRNA interactions. Other significant new features include (i) small insertions and deletions in miRNA seed regions and miRNA target sites, (ii) TargetScan context + score differences for assessing the impact of polymorphic miRNA–mRNA interactions and (iii) biological pathways. The browse and search pages of PolymiRTS allow users to explore the relations between the PolymiRTSs and gene expression traits, physiological and behavioral phenotypes, human diseases and biological pathways.

## INTRODUCTION

MicroRNAs (miRNAs) are small, ∼22 nt long noncoding RNAs that usually act as posttranscriptional regulators by binding to the 3′-untranslated regions (UTR) of mRNAs (1,2). The mechanism of miRNA targeting largely depends on a miRNA binding to a complementary target site. Sequence polymorphisms in either miRNAs or their target sites may affect this binding, impacting miRNA function and resulting in significant downstream effects on gene expression and higher-order phenotypes (3–9). miRNA-related polymorphisms, including both single-nucleotide polymorphisms (SNPs) (10–16) and small insertions and deletions (INDELS) (17,18), have been associated with many human diseases, including cancers (10–13,17), diabetes (14,18), Parkinson’s disease (15) and Alzheimer’s disease (16).

The PolymiRTS database was developed to systematically identify DNA polymorphisms in miRNAs and miRNA target sites (PolymiRTS), and elucidate their potential links to molecular, physiological, behavioral and disease phenotypes. The original version of the PolymiRTS database (19) focused on SNPs in putative miRNA target sites, as few miRNA target sites had been experimentally determined. However, in recent years, there has been both extensive use of existing experimental techniques and development of novel high-throughput methods that identify miRNA target sites, and we have expanded the PolymiRTS database to include these experimentally supported sites (20). While these experiments have greatly increased understanding of miRNA targeting, they did have limitations. Some high- (e.g. microarray) (21) and low-throughput (e.g. luciferase reporter assay) (22) experimental techniques determine miRNA–mRNA target pairs but do not provide specific binding locations, while CLIP-seq experiments (e.g. HITS-CLIP and PAR-CLIP) (23,24) identify specific binding locations within mRNAs but not the miRNAs that bind to the locations. Therefore, these experiments still required predictions based on sequence complementarity to determine either the specific binding location or the binding miRNA. In contrast, a recent advance in the high-throughput direct mapping of miRNA–mRNA binding sites from CLASH (cross linking, ligation and sequencing of hybrids) (25,26) experiments identifies both the miRNA and mRNA sequence simultaneously and presents the opportunity to identify polymorphisms in miRNA binding sites where both miRNAs and their binding sites are determined from the experiment. The newly available experimental data for miRNA–mRNA interactions and other rapidly growing genomic data allow us to perform a major update of the PolymiRTS database and make it a more useful and complete resource for linking polymorphic miRNA targeting to complex traits, diseases and biological pathways.

## NEW FEATURES

**Figure 1.**
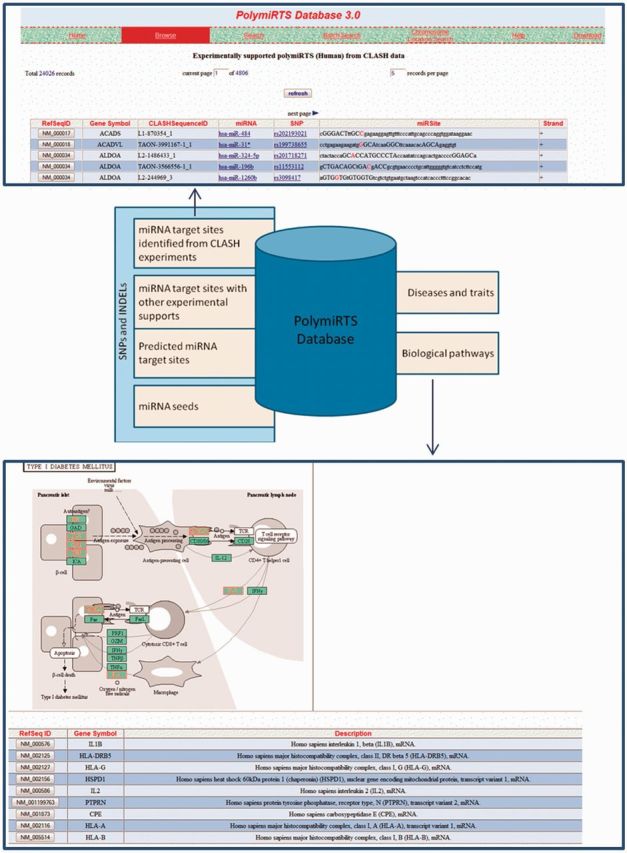
Overview of the PolymiRTS database contents and screenshots of two new functions: browsing miRNA targets identified by CLASH experiments and illustrating genes with polymorphic miRNA target site in the contexts of biological pathways.

We have expanded the PolymiRTS database with updated data and enhanced the database with new features, which include the following:

1. CLASH experiment data. CLASH is a new technique for high-throughput mapping of RNA–RNA interactions (25) that have recently been used for direct identification of miRNA–mRNA target pairs associated with human AGO1 protein (26), a component of the miRNA-induced silencing complex (miRISC). While previous methods for high-throughput identification of miRNA target sites (e.g. PAR-CLIP) identified only target site sequences and, then, relied on computational scans for complementary miRNA seeds to predict the targeting miRNAs, CLASH provides chimeric reads of miRNA and target site sequences and, therefore, directly identifies the binding miRNA and allows for improved determination of noncanonical miRNA–mRNA interactions, which involve bulged or mismatched nucleotides. The CLASH data set included in current PolymiRTS database contains 18 514 high confidence canonical and noncanonical target sites of 399 human miRNAs.
2. INDELs in miRNA sequences and miRNA target sites. About 18% of known human genetic variants are INDELs (27,28), constituting the second largest class of genetic variants after SNPs. Currently, >400 000 small INDELs have been identified in the human genome (27,28). Intuitively, INDELs may generally be more disruptive than SNPs in the alteration of miRNA targeting, as multiple nucleotides may be inserted in or deleted from the interacting sites. A recent genome-wide analysis of mutations in *Drosophila* provided evidences indicating that INDELs are more deleterious than SNPs in both coding and noncoding regions (29). Small INDELs altering miRNA sequences and target sites have been associated with human diseases (30). For example, a deletion in the target site of miR-657 has been associated with diabetes (18) and an insertion at miR-122 binding site has been linked to hepatocellular carcinoma (17). The PolymiRTS database now includes small INDELs (1–30 bases long) in miRNA target sites and miRNA sequences for both human and mouse (Table 1).
3. Using context + score to assess polymorphic miRNA–mRNA interactions. In a recent update, TargetScan (31) introduced the context + score for selection of the most favorable target sites for miRNAs. Context + score evaluates the binding of miRNAs to the context of entire 3′-UTR of a gene by summing over contributions made by individual sites that have perfect sequence complementarities to the miRNA seed (2–8 bases from the 5′ of mature miRNA sequences). In the PolymiRTS database, we have included differences in context + scores caused by polymorphisms in miRNA target sites and in miRNA seed regions. A more negative context + score difference indicates an increased likelihood that the target site is disrupted or a new target site is created by the derived allele.
4. Biological pathways. Biological pathways are widely used for illustrating the functional roles of genes and their interactions in biological processes. Polymorphisms in miRNA target sites and miRNA sequences may impact biological processes by affecting the posttranscriptional regulation of the target genes. For example, a SNP (rs5186) located in the binding site of miR-155 can change the expression of a target gene (AGTR1), which is associated with blood pressure (32). The updated PolymiRTS database establishes links between polymorphisms in miRNA target sites and their possible functional impact in biological processes by including gene pathways for human and mouse from the KEGG database (33). We highlighted the genes with polymorphic miRNA target sites in the context of pathways (Figure 1). A browse page lists the KEGG pathways with at least one gene that has an SNP or INDEL in a miRNA target site. Clicking on the Pathway ID shows a list of all genes in the pathway with polymorphic miRNA target sites.

**Table 1.** Summary of the updated contents of PolymiRTS database

Type of data			Number of records			
		Human		Mouse	
SNP	INDEL	SNP	INDEL
Polymorphisms in miRNA target sites	Identified by CLASH experiments	22 979	1047	NA	NA
	With other experimental supports^a^	1900	170	801	73
	Predicted from TargetScan	358 874	42 099	353 412	61 691
Polymorphisms in miRNA seeds		271	23	144	36
Genes with polymorphic miRNA target sites in different classes of biological pathways	Human diseases	1515	760	1752	162
	Cellular processes	1550	822	1469	97
	Environmental information processing	1779	1014	1714	96
	Genetic information processing	1050	425	1004	45
	Metabolism	1872	845	2313	150
	Organismal Systems	1847	969	2058	199
Genes associated with human traits in GWAS		4830		NA	

^a^PAR-CLIP, HITS-CLIP, microarray, pSILAC, reporter assay, western blot, etc.

## DATA COLLECTION AND DATABASE CONTENT

### Polymorphisms in miRNA target sites

SNPs and INDELs in 3′-UTRs of all RefSeq genes were collected using the ALL SNPs 137 (dbSNP build 137) track in the UCSC table browser (34). Specifically, we selected the following filter options: 3′-UTRs, SNP, insertion, deletion and INDEL for the mouse (mm10) and human (hg19) genomes. Mature miRNA sequences were downloaded from miRBase (release 20) and 3′-UTR sequences were downloaded from UCSC table browser. Perl codes for target prediction and context + score were downloaded from TargetScan (release 6.2) (31). We used the TargetScan context + score to assess the impact of polymorphism on miRNA–mRNA interaction. For each SNP and INDEL in the 3′-UTR of RefSeq genes, ancestral alleles were determined using pairwise sequence alignment data (hg19 and pantor2 for human, mm10 and rn4 for mouse) from the UCSC genome browser. Target site conservation was determined using the multiple sequence alignments data downloaded from Targetscan 6.2. The polymorphic miRNA target sites were assigned into four classes: ‘D' (the derived allele disrupts a conserved miRNA site), ‘N' (the derived allele disrupts a nonconserved miRNA site), ‘C' (the derived allele creates a new miRNA site) and ‘O' (other cases when the ancestral allele cannot be determined unambiguously). We calculated the differences in context + scores between the reference and derived alleles for each SNP or INDEL in the miRNA target sites. A more negative value of the context + score difference indicates an increased likelihood that the polymorphism significantly altered miRNA targeting of the sequence.

Based on the availability and the type of experimental supports for miRNA–mRNA interaction, miRNA target sites were classified as LT (binding is supported from a low-throughput experiment), HT (binding is supported by a high-throughput experiment), HTL (the specific binding location is supported by a high-throughput experiment), LTL (the specific binding location is supported by a low-throughput experiment) and N (no experimental support) (20). We have collected >14 000 new experimentally supported miRNA–mRNA interactions from two recently updated databases, miRecords (35) and miRTarBase (36). The PolymiRTS database now contains 2944 records for experimentally supported miRNA target sites with polymorphisms.

We mapped the miRNA target sites identified by CLASH experiments (26) to their genomic location using Ensembl (37). SNPs and INDELs in the genomic locations of the target sites were then collected from the dbSNP table in the UCSC genome browser. More than 18 000 miRNA binding sites from CLASH data were searched for polymorphisms. We found 24 026 of polymorphisms located in these miRNA target sites, including 22 979 SNPs and 1047 INDELs (Table 1).

### Polymorphisms in miRNA seed regions

Genomic locations of mature miRNAs were downloaded from miRBase (38). For each miRNA, we collected all SNPs and INDELs in the seed regions from dbSNP (39). We found 271 SNPs and 23 INDELs in human miRNA seeds, and 144 SNPs and 36 INDELs in mouse miRNA seeds. The mutant allele of each polymorphism disrupts the binding of the miRNA to its original target sites and may create new target sites. For each polymorphism in miRNA seeds and for each miRNA target, we computed the difference between context + scores for the mutant allele and reference allele.

### GWAS data and QTLs regulating molecular, physiological and behavioral phenotypes

The PolymiRTS database attempts to link polymorphisms in miRNAs and their target sites with variations in phenotypes and human diseases. These links may span multiple biological scales—from DNA sequence (QTLs) to gene expression and higher-order phenotypes (e.g. diseases). As described in the previous update (20), the PolymiRTS database includes GWAS data from NHGRI GWAS Catalog (40) and dbGaP (41), human expression QTL (eQTL) data from GTEx eQTL browser (42) and mouse QTL data from GeneNetwork (43). Since the last update, there has been a rapid growth of GWAS data. About 8000 newly available GWAS results were processed for the update and 1311 new records were added to the database.

### Biological pathways

We downloaded all KEGG pathways and then compared the list of genes in each pathway and genes with polymorphic target sites in the PolymiRTS database. We use the KEGG API interface to display the biological pathways. Figure 1 shows the ‘Type I diabetes’ pathway. Genes with polymorphisms in miRNA target sites were highlighted with ‘red’ font and ‘red’ box borders. There are two options for browsing biological pathways. The option ‘Experimental’ shows pathways with genes that have polymorphisms in experimentally supported miRNA target sites, including those identified by CLASH experiments. The option ‘ALL’ lists the pathways with genes that have polymorphisms in both predicted and experimentally supported miRNA target sites.

## DATABASE ACCESS

The PolymiRTS database can be accessed using browse and search interfaces. Different filtering options to set experimental types, target classes and conservation supports are provided for both the search and browse functions. The contents of the entire database are also available for download. Detailed information about the database access and usage is available in the help page of the PolymiRTS database.

## DISCUSSION

In the past few years, we have observed a significant increase of experimental miRNA–mRNA interaction data in the PolymiRTS database, and we expect this trend to continue in the future. The original version of the PolymiRTS (19) database (launched in 2006) included only SNPs in predicted miRNA target sites, as few experimentally identified miRNA target site were available. Since then, rapid advances in high-throughput technologies for detecting miRNA–mRNA interaction, such as PAR-CLIP (24) and HITS-CLIP (23), have made it increasingly important to include experimental miRNA binding data in the PolymiRTS database (20). However, while these experiments identify the locations of the binding sites, they still depend on computational algorithms to predict the binding miRNAs. Recently, a large number of miRNA target sites have been identified by the CLASH experiments (26), which is the first high-throughput technology that allows direct observation of miRNA–target pairs without the assistance of computational target prediction or scanning. CLASH data also includes a large number of noncanonical miRNA–mRNA interactions that cannot be easily identified by previous methods (26). Thus, the integration of CLASH data in the PolymiRTS database further reduced its dependence on miRNA target prediction, and therefore provides more complete and accurate information for polymorphic miRNA–mRNA interactions.

Recently, we created the SomamiR database (44) as a web-based platform for systematic investigation of the impact of somatic mutations on miRNA dysregulation in cancer. We also found that the integrated analysis of somatic and germ line mutations may provide useful insights on the functional impacts of both types of mutations (45). Therefore, we have created links between the entries of the two databases, allowing users to easily analyze both genetic polymorphisms and somatic mutations in miRNAs and their target sites.

The PolymiRTS database currently focuses on featuring polymorphisms in miRNA seed regions and miRNA target sites. Recent studies have revealed the functional importance of genetic polymorphisms in other parts of the miRNA regulome (46,47). Polymorphisms in pre-miRNAs, pri-miRNAs and miRNA promoters have been associated with many diseases. For example, a polymorphism in the pre-miRNA of has-miR-146a has been associated with the risk of cervical cancer (13), a polymorphism in the pri-miRNA of has-miR-128b has been linked to acute lymphocytic leukemia (48) and a polymorphism in the promoter of miR-200b-a-429 cluster has been associated with the risk of non-small cell lung cancer (49). Future updates of PolymiRTS database may include the SNPs and INDELs in pri-miRNAs, pre-miRNAs and promoters of miRNAs. The database also needs to be updated regularly to include the ever-growing new genomic data. We have developed a program package to process new data and add them to the database semi-automatically. This program package allows us to perform regular database updates more efficiently.

## FUNDING

Funding for open access charge: The University of Tennessee Health Science Center.

*Conflict of interest statement*. None declared.
